# Molecular Characterization of the 16S rRNA Gene of *Helicobacter fennelliae* Isolated from Stools and Blood Cultures from Paediatric Patients in South Africa

**DOI:** 10.4061/2011/217376

**Published:** 2010-11-29

**Authors:** Heidi E. M. Smuts, Albert Joseph Lastovica

**Affiliations:** ^1^Division of Medical Virology/National Health Laboratory Service, Department of Clinical Laboratory Sciences, University of Cape Town, Anzio Road, Observatory 7925, South Africa; ^2^Department of Biotechnology, University of the Western Cape, Modderdam Road, Bellville 7535, South Africa

## Abstract

Forty strains of *H. fennelliae* collected from paediatric blood and stool samples over an 18 year period at a children's hospital in Cape Town, South Africa, were amplified by PCR of the 16S rRNA. Two distinct genotypes of *H. fennelliae* were identified based on the phylogenetic analysis. This was confirmed by sequencing a portion of the beta subunit of the RNA polymerase (rpoB) gene. All isolates from South Africa clustered with a proposed novel *Helicobacter* strain (accession number AF237612) isolated in Australia, while three *H. fennelliae* type strains from the northern hemisphere, NCTC 11612, LMG 7546 and CCUG 18820, formed a separate branch. A large (355bp) highly conserved intervening sequence (IVS) in the 16S rRNA was found in all isolates. Predicted secondary structures of the IVS from the 16S rRNA and 23S rRNA were characterised by a primary stem structure formed by base pairing of the 3′ and 5′ ends and internal loops and stems. This phylogenetic analysis is the largest undertaken of *H. fennelliae*. The South African *H. fennelliae* isolates are closely related to an Australian isolate previously reported to be a possible novel species of Helicobacter. This study suggests that the latter is strain of *H. fennelliae*.

## 1. Introduction

Since the discovery of *Helicobacter pylori* (*H. pylori*) by Warren and Marshall in 1983 [1], more than 30 non-*pylori-Helicobacter *species have been described [2, 3]. To date,* H. bizzozeronnii, H. canadensi*s*, H. canis, H. cinaedi, H. fennelliae, H. felis, H. heilmannii*, *H. pullorum, H. rappini, H. salomonis, H. winghamensis, *and* H. westmeadii *have been found in humans with gastritis, enteritis, and septicaemia [3–8]. *H. fennelliae* was first described in 1985 as a new *Campylobacter* species isolated from asymptomatic homosexual men with enteritis and proctitis [9]. This organism was subsequently reclassified as a *Helicobacter* species based on 23S rRNA hybridisation studies [10]. *H. fennelliae* is a fastidious organism and difficult to culture; thus, there are very few reports of the clinical relevance of the organism. In 2000, Tee et al. [11] described a novel species of *Helicobacter *isolated from the blood of a young aboriginal child with diarrhoea and vomiting which was most closely related to *H. fennelliae*. The authors proposed, this may be a new species of *Helicobacter.*

From 1977 to 1990, the routine microbiological laboratories at Red Cross War Memorial Children's and Groote Schuur Hospitals in Cape Town, South Africa used a variety of antibiotic-containing media plates and standard microaerophilic atmospheric growth conditions for the isolation of *Campylobacter *and other *Epsilonproteobacteria*. *H. fennelliae* and other fastidious H_2_-requiring and antibiotic-sensitive *Epsilonproteobacteria* were never isolated under these conditions [12]. With the introduction of the “Cape Town protocol”, an isolation method that uses membrane filtration onto antibiotic-free plates and subsequent incubation in an H_2_-enriched microaerophilic atmosphere, *H. fennelliae* and other fastidious *Epsilonproteobacteria* were, and still are, routinely isolated from paediatric stool and blood cultures in Cape Town [12]. Over an 18-year period, since the introduction of this protocol in October 1990, *H. fennelliae* has been isolated from 5.6% (347/6249) stool samples from children with diarrhoea [8]. In addition, *H. fennelliae* was isolated from 15/174 (8.6%) paediatric blood culture samples negative for *Campylobacter* or other *Helicobacte*r species. This is highly suggestive of the fact that *H. fennelliae* may be a significant pathogen and is probably considerably underreported due to inadequate isolation methods [12]. 

Phenotypic and biochemical tests are usually used to identify bacterial isolates in the clinical setting. However, there are limitations to these assays, and thus sequencing and the phylogenetic analysis of the 16S rRNA are often utilised to identify new isolates. 

The access to a large number of *H. fennelliae* isolates from Cape Town provided the opportunity to look at the genetic diversity of these isolates and compare the data to that available in the GenBank database. The 16S rRNA and a portion of the RNA polymerase subunit B (rpoB) gene were analysed.

## 2. Materials and Methods

### 2.1. Bacterial Stains

Forty previously characterised strains of *H. fennelliae* collected from paediatric stool and blood samples over an 18-year period, from 1990–2008, were analysed in this study. Characterisation was performed using standard phenotypic and biochemical methods [12]. Clinical data is shown in Table 1. The *H. fennelliae* reference strain, NCTC 11612, was also included.

### 2.2. DNA Extraction

DNA was extracted using either the CTAB methodology [13] or a boiling method. Briefly, a couple of microbeads maintained at −80°C were removed and added to 100 *μ*l of distilled water. The beads were boiled for 5 minutes and the supernatant used in the PCR reaction.

### 2.3. PCR Amplification

The 16S rRNA was amplified using primers designed by Marshal et al. [14]. Initially a portion of the rpoB gene was amplified with primers designed by Lim et al. [15]. To improve phylogenetic resolution, an additional antisense primer (5′ TTGCATCATCATGCTCC) amplifying a larger region (704 bp) was designed, based on the sequence data of Kuhnert and Burnens [16]. Two microlitres of extracted DNA or boilate was added to a 50 *μ*l PCR mix consisting of 2 U Super-therm polymerase, 1x PCR reaction buffer, 1.5 mM MgCl_2 _(JMR Holdings, Kent, UK), and 200 *μ*M of each dNTP (Roche Biochemicals, Mannheim, Germany). The cycling conditions were 1 cycle 95°C 2 minutes, 40 cycles of 95°C 15 sec, 52°C 25 sec, and 72°C 35 sec followed by a final 7-minute extension cycle at 72°C. The PCR products were analysed by 2% agarose gel electrophoresis and visualised with UV irradiation after staining with ethidium bromide.

### 2.4. Sequencing and Phylogenetic Analysis

PCR products of the 16S rRNA and rpoB genes were purified (Qiagen, Hilden, Germany) and sequenced directly using the BigDye Terminator ver1.1 commercial kit (Applied Biosystems, CA, USA). The nucleotide sequences were aligned with known sequences from GenBank using the CLUSTAL-X software [17]. A neighbour-joining phylogenetic tree was constructed using the Treecon software program (version 1,3b) with 500 bootstrap resamplings [18].

### 2.5. IVS Secondary Structure

The secondary structure of the 16S rRNA intervening sequence (IVS) of *H. fennelliae* with the lowest free energy was predicted using mfold version 3.2 [19, 20]. An IVS has also been found in the 23S rRNA of *H. fennelliae* isolate CCUG 18820 (accession number AY596237) [21], and a comparison between the IVS of the 23S rRNA and 16S rRNA was made.

### 2.6. Accession Numbers

The accession numbers of the 16S rRNA sequences of *H. fennelliae* from this study are GQ867137-GQ867176. The rpoB sequence accession numbers are GQ867083-GQ867136.

## 3. Results

### 3.1. PCR Detection

**Figure 1:**
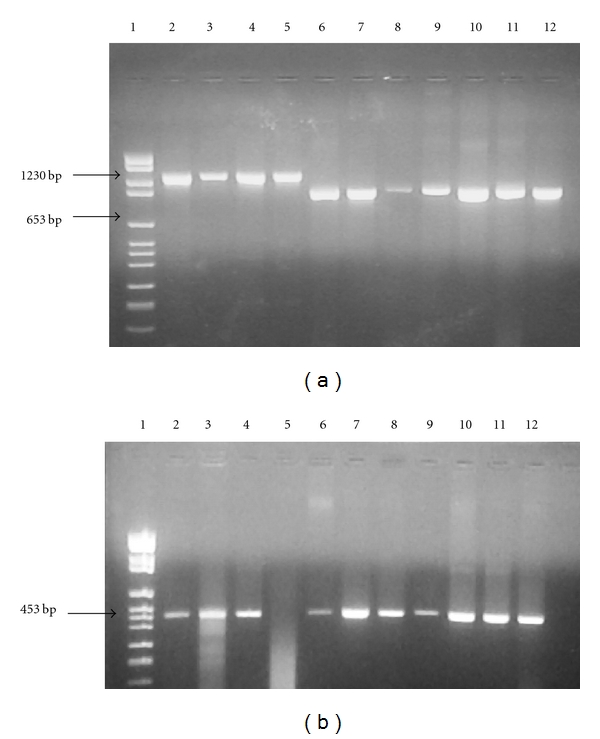
PCR amplicons of the (a) 16S rRNA gene of *H. fennelliae* (lanes 2–5) and other *Helicobacter* strains (lane 6: *H. cinaedi*, lanes 7-8: *H. pylori*, lane 9: *H. mesocricetorum*, lane 10: *H. pametensis*, lane 11: *H. cholecystus,* and lane 12: *H. canadensis*) and (b) rpoB gene of *H. fennelliae* (lanes 5–9) and other *Helicobacter* species (lane 2: *H. pylori*, lane 3: *H. cinaedi*, lane 4: *H. canadensis*, lane 10: *H. pullorum*, lane 11: *H. mustelae*, and lane 12: *H. cholecystus*).

The 16S rRNA of all 40 isolates of *H. fennelliae* were successfully amplified, yielding a 1340-bp product. This is larger than the other *Helicobacter* species due to the presence of IVS in *H. fennelliae*. A representative sample of 16S rRNA PCR products amplified from different *Helicobacter *clinical isolates is shown (Figure 1(a)). The rpoB gene was amplified from 32/40 (80%) isolates, with some examples shown (Figure 1(b)).

### 3.2. Clinical Information

Clinical and demographic data are shown in Table 1. The mean age of the 40 children from whom *H. fennelliae* was isolated was 14 months (range: 1–48 months). The male : female ratio was similar, 1.0 : 1.1. The majority of the children presented with symptoms of diarrhoea (31/40, 77.5%). *H. fennelliae* was isolated from blood cultures in 5 cases; 2 children had pneumonia, 2 had diarrhoea, and there was a single case of meningococcaemia. The immunological status of these children was not known. Dual infection with other enteric organisms was found in 21 samples (52.5%) of which the majority (19/21, 90%) were* Campylobacter* species, and in 2 samples *Shigella *and *Giardia* species were found. In the remaining 19 samples, *H. fennelliae* was the only organism cultured. The samples were not screened for viral enteric pathogens.

### 3.3. Phylogenetic Analysis

**Figure 2:**
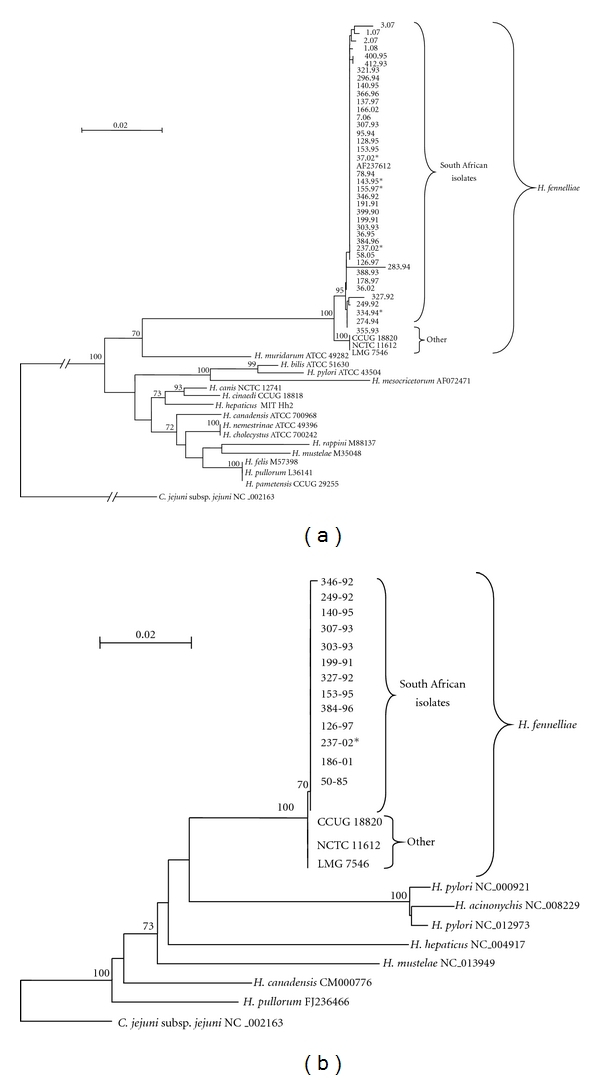
Phylogenetic trees of the 16S rRNA (a) and rpoB gene (b) of *H. fennelliae* and other *Helicobacter *species. ^*∗*^indicates blood culture samples.

Sequence analysis confirmed the identification of *H. fennelliae* isolates. The 16S rRNA phylogenetic tree was rooted with *Campylobacter jejuni *subsp.* jejuni* (accession number NC_002163) and showed two distinct branches of *H*. *fennelliae*. The isolates from South Africa all branched with an Australian *Helicobacter *strain (accession number AF237612), while those from Europe and USA formed a separate branch with a bootstrap value of 100% (Figure 2(a)). This branching pattern was not present using the smaller rpoB PCR fragment (data not shown), but on a limited number of *H. fennelliae *samples (*n* = 13), where the larger fragment was successfully amplified, the separation of the South African isolates from the other strains is observed (Figure 2(b)). A single nucleotide change (C-T) at position 295 of the 16S rRNA differentiated the types of strains NCTC 11612, LMG 7546, and CCUG 18820 from the South African isolates. The 16S rRNA sequence of isolate 283.94, although clustered with the South African *H. fennelliae* isolates, had a region of 57 bp (nt 1039–1096) with 10 nucleotide changes. BLAST analysis showed the sequence to have closest homology (97%) with the 16S rRNA of *Campylobacter hyointestinalis* subsp. *lawsonii *(accession number AB301965). *H. fennelliae* isolated from the blood did not cluster separately to those from stool samples (Figure 2(a)). The intraspecies variation of South African strains of *H. fennelliae *was 0.1–0.6% while between South African and the 3 type strains was 0.7–0.9%.

### 3.4. Intervening Sequence

All *H. fennelliae* isolates contained an IVS of 355 bp. The DNA sequence was inserted at nucleotide position 175 (based on *H. pylori *type strain NCTC 11637, accession number Z25741). The IVS sequences were identical to the type strains NCTC 11612, LMG 7546, and CCUG 18820 with the exception of isolates 327-92, 249-92, 355-93, 274-94, and 334-94. These 5 isolates had a C-T transition at position 307. The nucleotide composition of the region is A-T rich (64.5%). BLAST analysis showed that 122 bp of the IVS had a high similarity of 79% and 77% to the 23S rRNA of *H. canis* NCTC 12743 and *Helicobacter* sp. MIT 01-5592B, respectively, with an opposite polarity. In addition, there was a region of 70 bp which showed significant homology (90%) to *H. mesocricetorum* ATCC 700931. No open reading frames were noted.

**Figure 3:**
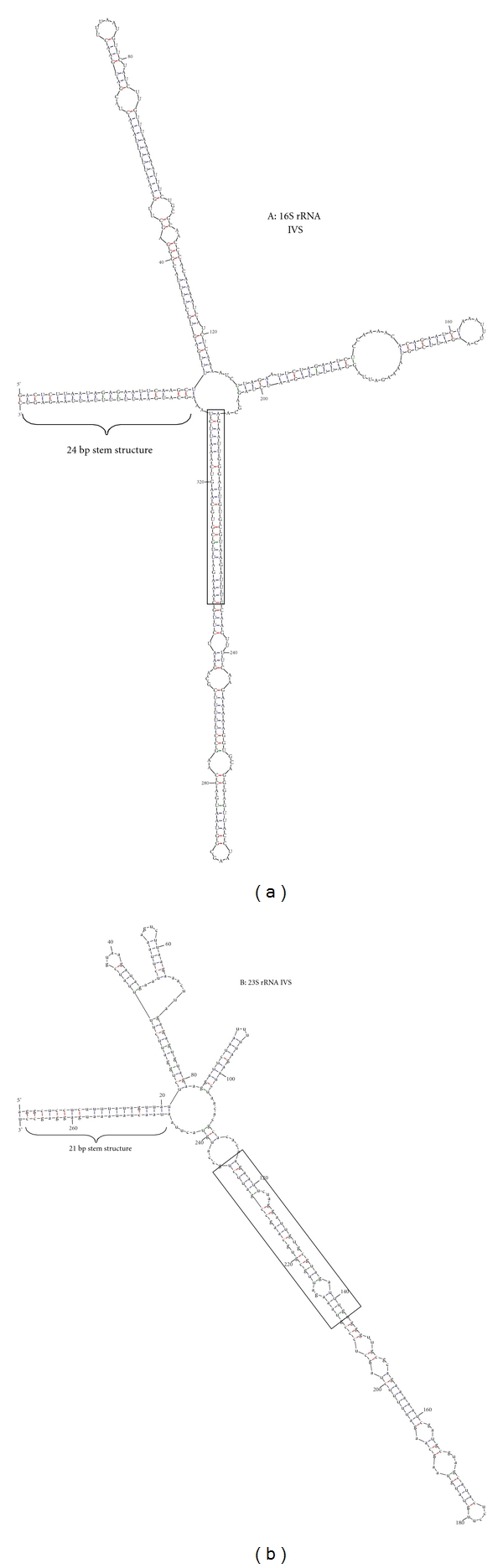
Predicted RNA secondary structures of the IVS from the 16S rRNA (a) and 23S rRNA (b) of *H. fennelliae.* Boxed area shows region of homology between the 2 IVS sequences.

The secondary structure of the 16S rRNA IVS predicted a conformation as seen in Figure 3(a) with free energies of −166.3 kcal.mol at 37°C. No suboptimal structures were found. The 5′ and 3′ ends of the 16S rRNA IVS were complementary resulting in a 24 bp stem structure. The 271 bp 23S rRNA IVS of *H. fennelliae *CCUG 18820 had a similar 21 bp stem structure (Figure 3(b)). A region of homology (26 bp) between the two IVS sequences was noted (boxed area Figure 3).

## 4. Discussion

This study identified 2 distinct genotypes of *H. fennelliae* based on the phylogenetic analysis of the 16S rRNA and rpoB genes. All isolates from South Africa clustered with the *Helicobacter *strain AF237612 isolated by Tee et al. [11], while the 3 isolates from the northern hemisphere, NCTC 11612, LMG 7546, and CCUG 18820, formed a separate branch with bootstrap value of 100%. Tee et al. [11] proposed that their novel *Helicobacter *strain may be a new species based on the 16S rRNA sequence analysis and a sequence similarity of ≤97% with *H. fennelliae* CCUG 18820 (accession number M88154) [22]. Our analysis of the latter sequence does not support this observation. The discrepancy between these observations can possibly be explained by the fact that the original M88154 sequence, deposited in GenBank in 1993 by Dewhirst and Paster [23], was subsequently replaced in 2004 by the same authors. This is after the paper by Tee et al. was published in 2000. In the earlier M88154 sequence, no IVS was present. Our results suggest that *Helicobacter* strain AF237612 is probably a *H. fennelliae* species. 

Sequencing of the housekeeping gene, rpoB, is increasingly being used to confirm 16S rRNA-generated phylogenetic trees and identify bacteria and closely related species in the clinical setting [24]. The taxonomic resolution of this gene is more than 3 times greater than that of 16S rRNA for a number of different bacteria, including *Vibrio*, *Bacillis,* and *Pseudomonas* [25–27]. In this study phylogenetic analysis of the larger rpoB fragment confirmed the separation of *H. fennelliae* isolates into 2 genotypes. 

Intervening sequences of variable lengths and sequences can be found in both the small (16S rRNA) and large subunit ribosomal RNA (23S rRNA) of many bacteria [28]. These form stable stem-loop secondary structures [28]. IVSs have been described in a number of *Campylobacte*r (*C. coli, C. curvus, C. fetus, C. helveticus, C. hyointestinalis, C. jejuni, C. sputorum, C. rectus, *and* C. upsaliensis,*) and *Helicobacter* (*H. bilis*, *H. canis*, *H. fennelliae, H. mustelae, *and* H. muridarum) *species [29–37]. The IVS found in the 16S rRNA of *H. fennelliae* isolates examined in this study is highly conserved. The A-T rich nature of this region is also preserved [28]. The C-T transition noted in 5 isolates did not alter the secondary structure of the IVS (data not shown). Although the IVS of the 23S rRNA is shorter than that of the 16S rRNA, there is a region of homology indicating a possible common ancestral element. The predicted secondary structures of both the 23S rRNA and 16S rRNA contained a stem of 21 bp and 24 bp formed, respectively, by the 5′ and 3′ inverted repeats. These may act as recognition sites for excision by RNAse III during rRNA maturation [28, 34]. 

Most *Helicobacter *species that cause diarrhoea can also be isolated from the blood [6]. *H. fennelliae* is infrequently reported as causing bacteraemia, and this may in part be due to the fastidious nature of the organism [11, 36, 38–40]. In this study 5 of the 15 *H. fennelliae *strains isolated from the blood were examined. There were no sequence differences between *H. fennelliae* strains isolated from blood or stool samples. 

In conclusion, this molecular and phylogenetic study is the largest undertaken of *H. fennelliae* with results indicating the presence of 2 genotypes. The South African isolates are more closely related to the Australian *Helicobacter* strain, a probable *H. fennelliae* species, isolated by Tee et al. [11] than to the 3 type strains.

## Figures and Tables

**Table 1:** Clinical and demographic details of the *H. fennelliae* isolates from paediatric stool and blood cultures.

Isolate	Age (months)	Sex	Clinical details	Stool	Coisolates
399.90	13	Male	Mucoid diarrhoea	L	C.jj1
191.91	30	Female	Eosinophilia	F	None
199.91	1	Female	Diarrhoea	W	*C.upsal*
249.92	9	Female	Chronic diarrhoea	W	*C.j doyl*
327.92	48	Female	Asthmatic	F	*C.upsal*
346.92	30	Male	FTT	F	*C.upsal*
303.93	18	Female	Asthma	F	*C.upsal*
307.93	12	Female	Chronic diarrhoea	F	C.j doyl,
					*C.upsal*
321.93	24	Female	Acute diarrhoea	L	*C.upsal*
355.93	31	Male	Loose stools	L	None
388.93	7	Male	Persistent diarrhoea	W	None
412.93	48	Male	Chronic diarrhoea	W	C.upsal
78.94	14	Female	Dysentery	L	C.jj1
95.94	14	Female	Chronic diarrhoea	L	C.conc
274.94	12	Male	Chronic diarrhoea	L	C.j doyl
283.94	6	Female	Chronic diarrhoea	L	None
296.94	7	Female	Diarrhoea and vomiting	W	None
334.94	?	Female	Blood culture		None
			Pneumonia, septicaemia		
36.95	24	Male	Periodic diarrhoea 2 months	L	*Giardia*
128.95	14	Male	Prolonged diarrhoea	L	None
140.95	13	Male	Dysentery	F	None
143.95	5	Male	Blood culture		None
			diarrhoea, acidotic		
153.95	15	Male	Kwashiorkor	W	*C.upsal*
400.95	30	Female	Dysentery, chronic diarrhoea	L	*C.upsal*
366.96	31	Male	Diarrhoea	L	None
384.96	12	Female	Diarrhoea and vomiting	L	None
126.97	11	Female	Gastroenteritis	W	None
137.97	18	Male	HIV+	L	*C.upsal*
155.97	13	Male	Blood culture		None
			meningococcaemia		
178.97	12	Female	Acute diarrhoea and vomiting	L	None
36.02	4	Male	Umbilical haematoma	L	C.jj1
37.02	6	Female	Blood culture		None
			gastroenteritis, FTT marasmus, fever		
166.02	22	Female	Gastroenteritis	W	*C.upsal*
237.02	8	Female	Blood culture		None
			pneumonia		
58.05	5	Male	Chronic diarrhoea	L	C.jj1
7.06	11	Female	Dysentery	W	None
1.07	33	Female	Diarrhoea	?	None
2.07	20	Male	Dysentery	?	None
3.07	15	Male	Diarrhoea	?	C.conc
					C.coli
1.08	22	Male	Diarrhoea	?	*Shigella *
					*sonnei*

Stools: L: loose, W = watery, F = formed, ?: not recorded. C. coli = *Campylobacter coli*, C.conc = *Campylobacter concisus,* C.jj1 = *Campylobacter jejuni *subsp*. jejuni* biotype 1, C.j doyl = *Campylobacter jejuni *subsp*. doylei, *C.upsal = *Campylobacter upsaliensis*. FTT = failure to hrive.
